# Correction: *STEMorph*: a set of morphed emotional face stimuli from angry to happy derived from *NimStim*

**DOI:** 10.3389/fpsyg.2026.2017143

**Published:** 2026-10-29

**Authors:** Mohammad Ebrahim Katebi, Mohammad Hossein Ghafari, Tara Ghafari

**Affiliations:** 1 School of Cognitive Sciences, Institute for Research in Fundamental Sciences, Tehran, Iran; 2 School of Medicine, Tehran University of Medical Sciences, Tehran, Iran; 3 School of Medicine, Shahid Beheshti University of Medical Sciences, Tehran, Iran; 4 Department of Experimental Psychology, University of Oxford, Oxford, United Kingdom; 5 Oxford Centre for Human Brain Activity (OHBA), Oxford Centre for Integrative Neuroimaging (OxCIN), Department of Psychiatry, University of Oxford, Oxford, United Kingdom

**Keywords:** facial emotional expressions, morphed stimuli, *NimStim*, *STEMorph*, stimulus set

In the published article, there was an error in Figure 2 as published. One of the example face stimuli shown in the figure depicted a model who had consented to the use of their face in the stimulus set but had not given consent for their face to be published. This face has been replaced with that of a model who has consented to publication. No other element of the figure has been changed. The corrected Figure 2 appears below.

**Figure 2:**
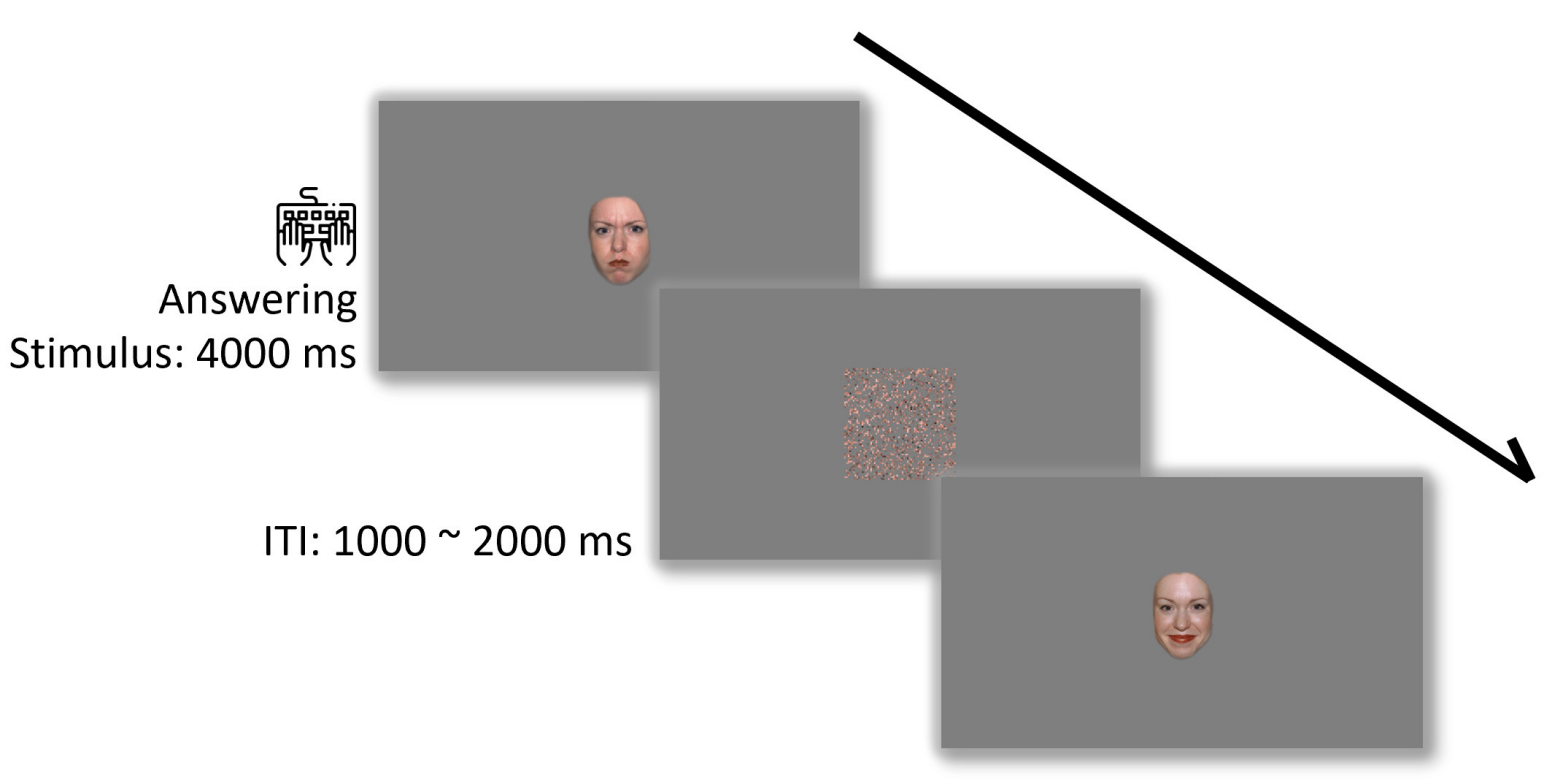
Schematic presentation of the experimental design. Stimuli appeared at the center of the screen, and participants rated the emotional intensity of each face within a 4,000ms viewing window without fixation restrictions. Each stimulus was followed by a randomized inter-trial interval (ITI) of 1,000–2,000ms, during which a noise mask was displayed. Facial images are adapted from the NimStim Set of Facial Expressions and were obtained directly from the Developmental Affective Neuroscience Lab under its terms of use (Tottenham et al., 2009).

The original version of this article has been updated.

