# Supplementary material for: STEMorph: a set of morphed emotional face stimuli from angry to happy derived from NimStim
Source: Front Psychol. 2026 Jul 29;17:1751625. doi: 10.3389/fpsyg.2026.1751625 (PMC13461392; doi:10.3389/fpsyg.2026.1751625)
Supplement: Supplementary file 1 [file Supplementary_file_1.docx]

**Supplementary Figure 1**

Face Identity Rating Profiles.

Note. A) Mean emotional ratings averaged across all nine morph steps for each of the 22 face identities, sorted in ascending order. Female faces are shown in teal and male faces in navy. Dashed horizontal lines indicate the overall mean for female and male faces separately. Identity labels on the x-axis report the person number and gender abbreviation (F = Female, M = Male). B) Distribution of mean emotional ratings for female and male face identities. Violin shape represents the kernel density estimate. Individual face identity means are overlaid as jittered points. Statistical comparisons: Mann–Whitney U test p-value (one-tailed, female > male), and Cohen’s d effect size. C) Mean emotional ratings for each of the 22 NimStim face identities (rows) across the nine morph steps (columns). Female faces (F, persons 1–11) are shown in the upper section and male faces (M, persons 12–22) in the lower section. Cell color reflects mean rating magnitude on a red–yellow–green scale.
